# Psychometric properties of the Negative Stereotypes Towards Aging Questionnaire (CENVE) among a sample of Portuguese adults

**DOI:** 10.1186/s41155-018-0085-0

**Published:** 2018-01-27

**Authors:** Cristina Nunes, Susana Menéndez, Cátia Martins, Helena Martins

**Affiliations:** 1 Centre for Research in Psychology – CIP-UAL, Lisbon, Portugal; 2 Department of Developmental and Educational Psychology, University of Huelva, Huelva, Spain; 3 University of Algarve, Faro, Portugal

**Keywords:** Aging, Assessment, Negative stereotypes, Validation

## Abstract

Stereotyped beliefs about old age and the aging process have proven to strongly promote negative behaviors toward the elderly, with unfavorable influences on their mental and physical health. Therefore, it is important to assess negative aging attitudes with brief but reliable and validated measurement instruments. The psychometric properties of the Portuguese version of the Negative Stereotypes Towards Aging Questionnaire (15 items self-reported) are explored and described in a sample of 302 participants (213 females and 89 males) between the ages of 18 and 59 years old. Confirmatory Factor Analysis shows a one-factor structure, similar to the Spanish version. The internal reliability and mean inter-item correlation showed satisfactory psychometric proprieties. Factorial ANOVA reveals that differences in agism beliefs are mainly related to educational level, with lower negative stereotypes in more educated adults. No differences were found concerning gender. This instrument may be a useful tool to assess negative stereotypes toward old age and the aging process.

## Background

Demographic aging is rapidly accelerating worldwide, and this shift has profound implications for today’s societies (World Health Organization, 2015). Although it is well known that the number of aging people is increasing with time, much less is known about what these individuals may have to deal with in the number of generalizations associated with the aging process. Stereotypes concerning aging have been shown to predict health, illness, and other behavioral outcomes in later life. Positive age stereotypes are associated with positive effects, whereas negative age stereotypes are related to negative effects (Meisner, 2012). Although there is a tendency for a more positive image and greater evidence supporting that older adults can contribute to society in many different ways, some pervasive misconceptions, attitudes, and stereotypes still prevail (Dionigi, 2015).

Stereotypes are unchallenged myths, overstated beliefs or tendentious, preconceived ideas, often negative, associated with a category that are widespread, and mainly used to define and limit people or groups of people in society (Fiske & Tablante, 2015; Helmes & Pachana, 2016). They have important influences on behavior and operate through a variety of mechanisms (Helmes & Pachana, 2016). Stereotypes of aging include assumptions and generalizations about older people without regard for individual differences or unique circumstances (Fiske & Tablante, 2015; Moreira, 2012; Wachelke & Contarello, 2011).

Agism is the stereotyping of and discrimination against individuals or groups based on their age (Swift, Abrams, Lamont, & Drury, 2017). It can take many forms, including prejudicial attitudes, discriminatory practices, or institutional policies and practices that perpetuate stereotypical beliefs (Smith, Bergeron, Cowart, Ahn, Towne, et al., 2016). Agism includes cognitive, behavioral, and emotional manifestations (Ayalon & Tesch-Römer, 2017) and reinforces social inequalities (Rippon, Kneale, de Oliveira, Demakakos, & Steptoe, 2014).

These negative stereotypes have serious consequences both for seniors and for society in general (World Health Organization, 2015). Agism and negative attitudes can influence the potential for healthy and active aging (Nelson, 2016; Rippon et al., 2014). In fact, stereotypes about aging have a significant influence on older people themselves and can deleteriously impact their health, reduce autonomy, independence, and quality of life (Swift et al., 2017). Research shows that older adults are often stereotyped as feeble, incompetent, sick and useless, disinterested and apathetic, socially isolated, depressed, rigid, out of touch, burdensome, dependent, and cognitively impaired (Cook, 2011). For older people who accept and incorporate these stereotypes, negative self-perceptions of their aging process may hinder them (Lamont, Swift, & Abrams, 2015; Sánchez, Trianes, & Blanca, 2009).

Several studies have stated individual differences about agism. Regarding sex differences, research has noted that women have less ageist attitudes compared to men (Bodner, Bergman, & Cohen-Fridel, 2012). Concerning age, researchers have found that there is a changing pattern across the life span; a study of Bodner et al., (2012) noted that these types of attitudes change as people age as they found that a middle-age group revealed higher negative levels when compared to younger and older participants.

Given the practical implications of this issue, in the last few decades, many researchers have analyzed the implications of agism and negative stereotypes, with specific regard to negative influences on the mental and physical health of older people (Ayalon & Tesch-Römer, 2017; Dionigi, 2015; Nelson, 2016; World Health Organization, 2015). Several investigations note a clear and direct threat to the cognition of the elderly, namely, when they believe and incorporate these negative stereotypes themselves (Marquet, Missotten, & Adam, 2016; Nelson, 2016). For example, stereotypes about age-related cognitive decline may have a detrimental effect on older adults regarding their cognitive performance. Whether they are consciously or unconsciously perceived by the elderly, beliefs in stereotypes can operate through various mechanisms such as increased anxiety and the adoption of a vigilant attitude (Marquet et al., 2016). Research also shows that the will to live is decreased, and seniors are less interested in engaging in healthy preventive behaviors. Negative age stereotypes also have significant effects on the physical well-being of older persons. Recovery from illness is impaired, cardiovascular reactivity to stress is increased, and longevity is decreased (Nelson, 2016).

These negative stereotypes have important social and economic implications (Lamont et al., 2015), which are particularly relevant given the aging population. Stereotypes can be a barrier to developing good policies and can seriously impact the quality of health and social care that older people receive.

Despite the importance of this issue, instruments to assess negative stereotypes about old age are scarce. Polizzi (2003) notes that difficulties persist in making an accurate assessment of attitudes toward the elderly. According to this researcher, the Aging Semantic Differential, one of the most widely used instruments to assess the stereotypic attitudes toward older adults, developed by Rosencranz and McNevin (1969) employs adjectives not necessarily descriptive of attitudes today and is outdated. In the same sense, some weaknesses in the Kogan Attitudes Toward Old People Scale developed by Kogan (1961) are also identified, and the need for further refinement is suggested due to the failure associated with two of its subscales (Iwasaki & Jones, 2008).

From previous research, we can also mention the questionnaire Facts on Aging Quizzes (FAQ 1 and FAQ 2) developed by Palmore (1988). This questionnaire has been used in some studies. However, researchers have suggested that given the areas addressed (e.g., physical, mental, and social facts) and its reliability, these instruments may not be suited for evaluative use (Snyder & Tyler, 2001).

These identified fragilities justify the relevance of the Negative Stereotypes Towards Aging Questionnaire (Cuestionario de Evaluación de Estereotipos Negativos Hacia la Vejez – CENVE) developed by Blanca, Sánchez, and Trianes (2005). This questionnaire is based on the Palmore Questionnaire (Palmore, 1988) and the Montorio and Izal questionnaire (Montorio & Izal, 1991). It consists of 15 items in the original form, corresponding to negative stereotypes of old age, ranging from 1 (strongly disagree) to 4 (strongly agree). The first psychometric study was conducted with a sample of 757 noninstitutionalized elderly between the ages of 65 and 96 years old. The analysis showed three factors (health, motivational-social, and character-personality), which can vary between 5 and 20 points with higher scores indicating a high degree of belief in negative stereotypes about old age and low scores indicating a weak belief in negative stereotypes regarding old age.

To validate the questionnaire with nonelderly subjects, a second study was conducted by Menéndez, Cuevas-Toro, Pérez-Padilla, and Lorence (2016) with a total of 350 adults under the age of 65. The results did not confirm the original factorial structure in three factors, suggesting a one-dimensional structure.

To adapt the CENVE to the Portuguese population, an exploratory study was conducted by Authors (2015) with a sample of 240 adults between the ages of 20 and 59 years old. The factor analysis conducted did not reveal the same structure as the original version (Blanca et al., 2005). Our results confirm a one-dimensional solution presented by Menéndez et al. (2016) to assess the negative stereotypes related to behavior and the physical and psychological health of older adults. All of the items were important for consistency and reliability.

Increasing our awareness about stereotypes and age discrimination is fundamental to developing appropriate policies and strategies for future interventions to combat agism. The aims of the current study are to analyze (1) the psychometric properties of the Portuguese CENVE version, including using Confirmatory Factor Analysis to examine its factor structure, and (2) the differences in negative aging stereotypes concerning sociodemographic characteristics.

## Method

### Participants

The sample was composed of 302 adults between the ages of 18 and 59 (*M* = 37.11, *SD* = 12.31), mostly women (70.65%) and most without any previous aging training (92.41%). Concerning their educational level, 25.69% completed primary school, 42.71% completed high school, and 31.60% had university studies. Most of the participants were employed (64.79%) or students (20.42%), 49.48% were married or living together, and 39.45% were single.

### Instrument

To evaluate the negative aging stereotypes, we used the Portuguese version of the CENVE (*Cuestionario de Evaluación de Estereotipos Negativos Hacia la Vejez*; Blanca et al., 2005), composed of 15 items, ranging from 1 (strongly disagree) to 4 (strongly agree). The original version was structured in three dimensions of old age stereotypes referring to health (5 items, e.g., “Cognitive impairment—memory loss, disorientation, confusion, etc.—is an inevitable part of aging”), motivation (5 items, e.g., “Older people have fewer friends than younger people”), and personality (5 items, e.g., “As people get older they become more rigid and inflexible”). Higher scores reveal a more negative aging stereotype. This instrument is free access; therefore, no authorization was requested to original authors.

Participants also provided data concerning their sociodemographic characteristics: age, gender, educational level, work status, marital status, and previous aging training.

### Procedures

The original translation of the CENVE into the European Portuguese language was previously conducted (Authors, 2015). During the translation and retroversion of the RPQ, appropriate procedures were followed. The cultural adaptation was particularly considered, taking into account clarity, common language use, and conceptual equivalence of the scale. The questionnaire was then independently back-translated into Spanish. The original and the back-translated items were compared for non-equivalence of meaning, and items were revised when any discrepancies in meaning were detected until no semantic differences were identified between the Spanish and the Portuguese versions. Using a snowball sampling technique, graduate students from the Psychology Department of the University of Algarve were contacted and asked to participate in this study by answering and recruiting five adults to the CENVE.

### Data analysis

The data were analyzed using SPSS (version 22; IBM Corp. released 2013. IBM SPSS Statistics for Windows, version 22.0. Armonk, NY) and EQS 6.3 (Bentler & Wu, 2015). The factor structure of the CENVE Portuguese version was assessed with Confirmatory Factor Analysis (CFA) performed in EQS 6.3 (Bentler & Wu, 2015; Byrne, 2006) with the robust estimation methods. We calculated the goodness of fit indexes, including Satorra-Bentler chi-square/degrees of freedom (S-B*χ*2/df), incremental fit index (IFI), comparative fit index (CFI), and root mean square error of approximation (RMSEA). A chi-square/degrees of freedom value < 5 was considered acceptable, a value of 2 was considered good, and 1 equaled very good (West, Taylor, & Wu, 2012). An IFI/CFI ≥ .90 and RMSEA ≤ .10 indicated adequate fit, whereas an IFI/CFI ≥ .95 and RMSEA ≤ .06 indicated a good model fit (Byrne, 2006).

The CFA was performed on the original scale items using a covariance matrix. Modification indexes were considered to determine if any suggestion of model modification would significantly improve the measurement model. Items with a standardized loading above .30 were retained because factor loadings are typically considered to be meaningful when they exceed that value. Cronbach’s alpha values above .70 were considered to be good, mean inter-item correlations were considered good if they were between .15 and .50, and corrected item-total correlations were considered satisfactory if they were above .20 (Nunnally & Bernstein, 1994).

To analyze differences associated with the main and interaction effects of group characteristics, a factorial ANOVA was performed using CENVE scores as dependent variables and age, gender, and educational level as fixed factors. As factorial ANOVA requires that independent variables are qualitative, 33 and 66 age percentiles were computed to establish three equivalent groups.

## Results

Our first step in assessing the psychometric properties of the Portuguese version of CENVE was to replicate it using a CFA operating with the maximum likelihood (ML) method and the unifactorial structure proposed for this instrument by Menéndez et al. (2016) and Authors (2015). We were able to find support in all the goodness of fit indexes for the proposed one-factor model (Table 1).

**Table 1:** Goodness of fit indices for different ML models of CENVE

CENVE	S-B *χ*^2^	df	S-B *χ*^2^/df	IFI	CFI	RMSEA	Confidence interval (90%)
One-factorial model	203.42	90	2.26	.91	.91	.06	.05–.08

*ML* maximum likehood, *S-Bχ*^*2*^ Satorra-Bentler chi-square, *df* degrees of freedom, *IFI* incremental fit index, *CFI* comparative fit index, *RMSEA* root mean square error of approximation

Table 2 shows descriptive and metric analysis; all items had averages greater than 2 (with the exception of item 15: *M* = 1.70, *SD* = 0.84) revealing values in the upper levels. Concerning the distribution, the results are mostly negative and near zero, revealing a left and platykurtic tendency, but near normal.

**Table 2:** Descriptive and metric analysis of CENVE items

Items	*M*	SD	*Skewness*	*Kurtosis*	Corrected item-total correlation	*α* if item deleted	Item loadings
Item 1	2.57	0.85	− 0.45	− 0.48	.40	.86	.46
Item 2	2.31	0.88	0.08	− 0.76	.47	.86	.53
Item 3	2.56	0.91	− 0.10	− 0.78	.56	.85	.65
Item 4	2.05	0.96	0.54	− 0.69	.53	.85	.64
Item 5	2.11	1.06	0.46	− 1.07	.44	.86	.52
Item 6	2.38	0.91	− 0.04	− 0.86	.49	.86	.58
Item 7	2.61	0.89	− 0.19	− 0.68	.56	.85	.64
Item 8	2.36	0.94	0.01	− 0.94	.51	.86	.59
Item 9	2.98	0.89	− 0.61	− 0.33	.48	.86	.56
Item 10	2.70	0.90	− 0.19	− 0.74	.61	.85	.70
Item 11	2.77	0.88	− 0.40	− 0.50	.55	.85	.65
Item 12	2.58	0.94	− 0.20	− 0.84	.56	.85	.66
Item 13	3.15	0.80	− 0.79	0.29	.48	.86	.56
Item 14	2.03	0.90	0.65	− 0.31	.45	.86	.51
Item 15	1.70	0.84	0.92	− 0.15	.49	.86	.57

The Cronbach’s alpha revealed a good standardized coefficient (*α* = .86) and also the ordinal alpha (*α*_ordinal_ = .89), with all the items being important to total reliability. The item-total correlation was mostly above *r* > .45, showing a good discriminability of the scale items. All items had loadings above .45.

Table 3 summarizes the results of factorial ANOVA 2 (gender) × 3 (age group) × 3 (educational level). The corrected model obtained a statistical significant result explaining 13.3% of the variance but with moderate effect. Educational level was the only factor with a significant main effect. The Bonferroni post hoc test showed that participants with a primary educational level reported higher means of negative aging stereotypes (*M* = 39.72, *SD* = 7.45) than high school (*M* = 36.70, *SD* = 7.17) and university levels (*M* = 34.32, *SD* = 8.69).

**Table 3:** Relation between sociodemographic characteristics and CENVE scores

	*F*	*p*	Eta-partial^2^
G	1.49	.223	.01
AG	0.01	.987	.00
EL	3.77	.024	.03
G x AG	0.62	.537	.01
G x EL	0.01	.987	.00
AG x EL	1.49	.205	.02
G x AG x EL	2.39	.051	.03
*R* ^*2*^	.13		
G	*M*	*SD*	
Women	36.21	7.96	
Men	38.05	7.83	
AG			
< 29 years	34.82	7.98	
30–43 years	36.86	7.22	
> 44 years	38.46	8.25	
EL			
Low	39.72	7.45	
Medium	36.70	7.17	
High	34.32	8.69	

*G* gender, *AG* age group, *EL* educational level

## Discussion

The aim of this study was to contribute with an analysis of structural validity and internal consistency of the Portuguese CENVE version. Therefore, a CFA was conducted to confirm the one-factorial structural proposed by Menéndez et al. (2016) and Authors (2015). Our results provide additional evidence for a one-factorial model, finding support in all the goodness of fitness indexes. The item loadings were above .51, except item 01 (*r* = .46), and the item-total correlation was mostly above *r* > .40, showing a good discriminability of the scale items (Nunnally & Bernstein, 1994).

Concerning the scores obtained in each level of negative aging stereotypes, the participants revealed punctuations situated in an intermediate position similar to Blanca et al. (2005) and slightly above Menéndez et al. (2016). All of the items were important to the consistency and reliability. The internal consistency analysis revealed satisfactory values, with all scores exceeding the recommended minimum Cronbach’s alpha of .70 (Nunnally & Bernstein, 1994).

The factorial ANOVA performed between educational levels showed differences regarding aging stereotypes. Participants with primary educational levels reported higher means of negative aging stereotypes than high school and university levels. This result is comparable with the findings of Menéndez et al. (2016) and highlights the relevance of formal education in the changing of aging attitudes (Sousa, Cerqueira, & Galante, 2008). In fact, educational level was the only discriminant sociodemographic characteristic of aging stereotypes. Contrary to other studies (Menéndez et al., 2016), neither age nor sex (Laditka, Fisher, Laditka, & Segal, 2004) was significant.

From the results explored above, this instrument can be a useful tool in the assessment of negative stereotypes not only in a research scenario but also in an interventions context (e.g., programs to promote positive beliefs toward aging).

Some limitations concerning this research must be mentioned. First, due to the inexistence of adequately validated instruments, Portuguese CENVE convergent validity assessment was not possible. It is, therefore, necessary to conduct more validation studies of alternative measures of aging stereotypes among Portuguese samples. Second, due to the cross-sectional nature of the study, some psychometric properties could not be evaluated (e.g., test–retest reliability). Additionally, in the future, other analysis including discriminant measures must be conducted to improve negative stereotypes research.

## Conclusions

Since research has reported that negative aging stereotypes can constitute barriers to the functionality of older adults and negatively influence their social status (Palmore, 1988), professionals must develop interventions that aim to change attitudes and beliefs starting at a young age. One must be aware that agism can take many forms (e.g., prejudicial attitudes, discriminatory practices, or institutional policies) and practices, perpetuating stereotypical beliefs (Smith et al., 2016). Therefore, interventions that are aimed at cognitive, behavioral, and emotional components (Ayalon & Tesch-Römer, 2017) are important, and CENVE can be a useful tool to assess the levels of negative stereotypes before and after intervention.
